# The application of radiomics in esophageal cancer: Predicting the response after neoadjuvant therapy

**DOI:** 10.3389/fonc.2023.1082960

**Published:** 2023-04-06

**Authors:** Hai Guo, Hong-Tao Tang, Wen-Long Hu, Jun-Jie Wang, Pei-Zhi Liu, Jun-Jie Yang, Sen-Lin Hou, Yu-Jie Zuo, Zhi-Qiang Deng, Xiang-Yun Zheng, Hao-Ji Yan, Kai-Yuan Jiang, Heng Huang, Hai-Ning Zhou, Dong Tian

**Affiliations:** ^1^ Department of Thoracic Surgery, West China Hospital, Sichuan University, Chengdu, China; ^2^ Department of Thoracic Surgery, Sichuan Tianfu New Area People’s Hospital, Chengdu, China; ^3^ College of Clinical Medicine, North Sichuan Medical College, Nanchong, China; ^4^ College of Medical Imaging, North Sichuan Medical College, Nanchong, China; ^5^ Department of General Thoracic Surgery, Juntendo University School of Medicine, Tokyo, Japan; ^6^ Department of Surgery, Tohoku University Graduate School of Medicine, Sendai, Japan; ^7^ Department of Thoracic Surgery, Suining Central Hospital, Suining, China

**Keywords:** esophageal cancer, neoadjuvant therapy, radiomics, radiology, prediction model

## Abstract

Esophageal cancer (EC) is one of the fatal malignant neoplasms worldwide. Neoadjuvant therapy (NAT) combined with surgery has become the standard treatment for locally advanced EC. However, the treatment efficacy for patients with EC who received NAT varies from patient to patient. Currently, the evaluation of efficacy after NAT for EC lacks accurate and uniform criteria. Radiomics is a multi-parameter quantitative approach for developing medical imaging in the era of precision medicine and has provided a novel view of medical images. As a non-invasive image analysis method, radiomics is an inevitable trend in NAT efficacy prediction and prognosis classification of EC by analyzing the high-throughput imaging features of lesions extracted from medical images. In this literature review, we discuss the definition and workflow of radiomics, the advances in efficacy prediction after NAT, and the current application of radiomics for predicting efficacy after NAT.

## 1 Introduction

Esophageal cancer (EC) is one of the most common cancers worldwide, ranking seventh in incidence and sixth in its overall mortality rate (1). The prognosis after EC is unsatisfactory, with a 5-year survival rate of approximately 25% (2). Although surgery has been regarded as an effective treatment for EC, the higher postoperative mortality and recurrence rate have prompted the investigation of multimodal treatments such as neoadjuvant therapy (NAT) (3). Currently, NAT combined with surgery has become the standard treatment for patients with locally advanced EC and is more effective in improving patient survival than surgery alone (4–7).

However, the prognosis of patients with NAT varies due to individual differences. For instance, the differences between esophageal squamous cell carcinoma (ESCC) and esophageal adenocarcinoma (EAC), and inconsistencies in the standard therapy for NAT, such as the use of radiotherapy compared to chemotherapy, pose a significant obstacle to achieving good outcomes (8–10). In addition, ypTNM and tumor regression grade (TRG) are used to evaluate the efficacy of NAT in EC patients (11, 12). Though the methods described above are being studied and proven to have a good effect on evaluating the prognosis of EC, several limitations remain (13, 14). Numerous researchers contend that in EC patients receiving NAT, the ypTNM stage mainly loses its prognostic significance and may differ from nation to nation (15–17). Meanwhile, there is still debate about the optimal TRG system, which restricts its application (18). Therefore, accurate prediction of outcomes in patients with EC after NAT is still necessary, and breakthroughs are urgently needed. Most recently, investigators have focused on novel applications such as radiomics to improve the patient pathway.

Radiomics is a non-invasive technique that involves the extraction of quantitative features from medical images, the selection of features by using particular methods, and the analysis correlating with clinical data for classification or prediction (19, 20). Our earlier research used radiomics to predict pathological and survival outcomes in patients with thymic epithelial tumors and to detect lung allograft rejection in a rat lung transplantation model, both of which demonstrated the effectiveness of radiomics in the prognostic analysis of cancer or lung transplantation (21, 22). Other previous studies have shown that radiomics can play an active role in the clinical staging, outcome assessment, and prognostic analysis of cancer. A systematic review on the value of radiomics in predicting response to treatment in patients diagnosed with gastrointestinal tumors showed that radiomic models and individual radiomic features enabled better prediction (area under the curve (AUC) or accuracy > 0.75) in 37 studies (23). In EC, radiomics can predict adverse events after NAT, thus allowing physicians to judge other treatment strategies for their patients. It has been demonstrated that radiomics better predicts pathological responses such as pathological complete response (pCR), complications, recurrence, and survival (**Table 1**) (24, 34, 35, 39–43).

**Table 1:** Predictive performances and application of radiomic models in esophageal cancer.

Outcomes	Imaging modalities	Number of patients	Prospective	Multi-center	Modeling methods	Predictive Performances^*^	Application	Reference
pCR								
	CT	55	No	No	LASSO	AUC, 0.86	Prediction of pCR in ESCC after nCRT	(24)
	CT	231	No	No	LR, SVM, KNN, NB, DC, RF, XGboost	AUC, 0.852	Prediction of pCR to nCRT in ESCC	(25)
	PET/CT	73	No	No	LASSO	AUC, 0.81	Prediction of response to nCRT in EC	(26)
	PET/CT	91	Yes	No	LASSO	AUC, 0.78	Prediction of response to nCRT in EC	(27)
	PET/CT	20	No	No	SVM, LR	AUC, 1.00	Modeling pathologic response of EC	(28)
	MRI	24	Yes	No	_	AUC, 0.914	Optimal timing for prediction of pCR to nCRT in EC	(29)
	MRI, PET/CT	54	No	No	LR	AUC, 0.914	Assessment of the response to nCRT in locally advanced ESCC	(30)
	PET/CT	96	No	No	LR	AUC, 0.857	Prediction of response to nCRT in EC	(31)
Recurrence								
	PET/CT	44	No	No	_	_	Prediction of recurrence and mortality of locally advanced EC patients	(32)
	PET/CT	44	No	No	_	_	Improvement of prognostic stratification in patients with ESCC treated with nCRT and surgery	(33)
	PET/CT	68	No	No	LR	AUC, 0.87 ± 0.06	Prediction of pCR and loco-regional control following nCRT in EC	(34)
	CT	206	No	No	LASSO	C-index, 0.746	Prediction of postoperative recurrence in patients with ESCC who achieved pCR after nCRT followed by surgery.	(35)
Survival								
	CT	239	No	Yes	RF	AUC, 0.69	Prediction of 3-year overall survival following chemoradiotherapy of EC	(36)
	CT	307	No	No	LASSO	C-index, 0.700	Improvement of survival prediction in ESCC	(37)
	PET/CT	65	No	No	RF	AUC, 0.822 ± 0.059	Prediction of treatment response and survival in EC patients treated with chemo-radiation therapy	(38)
	MRI, PET/CT	69	Yes	Yes	_	C- index, 0.82	Preoperative prediction of pathologic response to nCRT in patients with EC	(39)

*Only the best prediction outcomes were chosen for use with various modeling methods. pCR, pathological complete response; AUC, area under the curve; LASSO, least absolute shrinkage and selection operator; DC indicates decision tree; KNN, k-nearest neighbors; LR, linear regression; NB, naive bayes; RF, random forest; SVM, support vector machine; XGboost, extreme gradient boosting; ESCC, esophageal squamous cell carcinoma; nCRT, neoadjuvant chemoradiotherapy; EC, esophageal cancer.

Nevertheless, there are still some problems with the prediction and practical application of radiomics to EC patients receiving NAT, such as the dilemma of individual precision therapy, the controversy of surgical removal versus organ preservation after NAT, and some other pitfalls. This article will review radiomics in predicting response after NAT in EC, aiming to assist physicians in their decision-making for treatment strategies. To the best of the authors’ knowledge, this is the first literature review on applying radiomics in EC patients after NAT.

## 2 Radiomics

### 2.1 Brief introduction to radiomics

Radiomics is a high-throughput and non-invasive technique developed by Lambin et al. in 2012 to extract numerous imaging features from radiographic images that are hardly visible to radiologists. It further correlates these data with clinical outcomes like treatment efficacy, survival, or toxicity to develop identification or prediction models using objective methods (19, 20). It cannot be established without the development of medical imaging. Lambin et al. summarized the relationship between the development of medical imaging techniques and radiomics in the following four points: 1) innovations in medical devices (hardware), 2) innovations in imaging agents, 3) a standardized protocol allowing quantitative imaging, and 4) innovations in imaging analysis (19, 44). Radiomics can use high-dimensional data generated from medical imaging, such as computed tomography (CT), magnetic resonance imaging (MRI), positron emission tomography (PET), and the combination of PET and CT (PET/CT), to provide mathematical quantification of tumor phenotypes through radiomic features, and establish identification or prediction models to correlate with tumor characteristics, clinical results and specific gene-expression patterns (23, 45, 46). It can capture the heterogeneity within the tumor, which is affected by many factors such as intracellular factors or cell microenvironment, and is the main obstacle to the practical and individualized treatment of tumors. Thus, it guides clinical diagnosis, such as continuing surgery or retaining organs (20, 47, 48). However, radiomics is still a very young and exploratory field. Most established models have not been used for routine clinical treatment, and there is a lack of sizeable external validation (49). The disciplines behind it may still seem immature because of the inconsistent standards, heterogeneous methods, and quality control, which often does not exist (50, 51). In summary, as an emerging field, radiomics has excellent potential to improve health care, mainly providing a solid foundation for clinicians or radiologists to develop cancer treatment strategies. However, its clinical application and value still need further research and exploration due to some limitations and problems.

### 2.2 The workflow of radiomics

Although there are many technical methods of radiomics, its workflow is roughly divided into the following five parts: data selection, segmentation, feature extraction, feature selection, as well as modeling and validation (20, 44, 46, 52).

**Figure 1:**
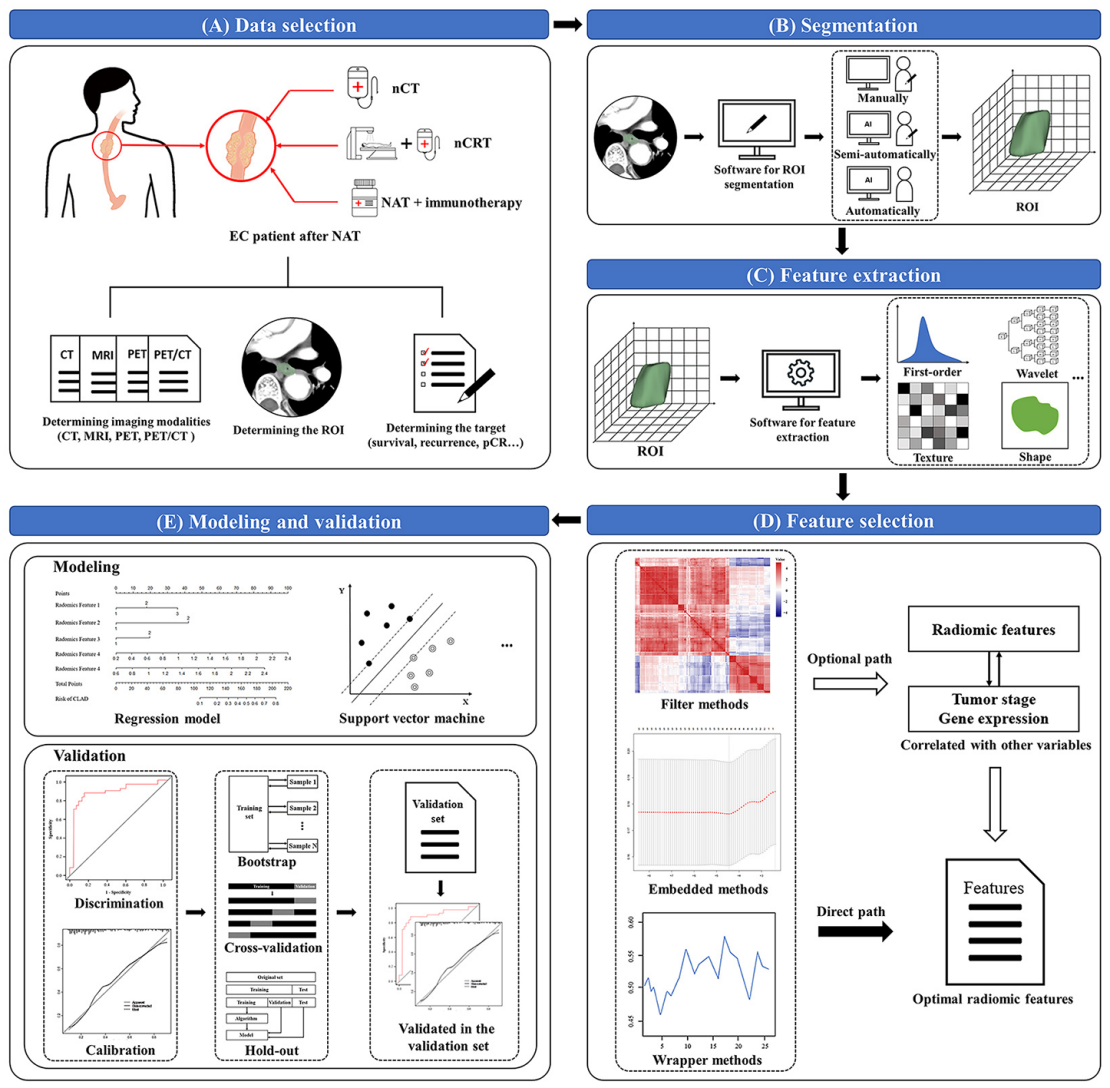
Workflow of radiomics. **(A)** Data selection: determines the imaging modalities, the tumor regions of interest (ROI), and a prediction target; **(B)** Segmentation: segments the delineated tumor ROIs in the original or processed images; **(C)** Feature extraction: extracts quantitative radiomic features through software or package from the tumor ROIs; **(D)** Feature selection: selects the extracted features by using the filter, embedded or wrapper methods; **(E)** Modeling and validation: models the selected radiomic features by specific methods, then discriminates and calibrates through bootstrap, cross-validation or hold-out methods. EC, esophageal cancer; NAT, neoadjuvant therapy; nCT, neoadjuvant chemotherapy; nCRT, neoadjuvant chemoradiotherapy; ROI, regions of interest; pCR, pathological complete response.

The first step in radiomics is determining the imaging modalities, the tumor regions of interest (ROI), and a prediction target. Second, we manually, semi-automatically or automatically segment the delineated tumor ROIs in the original or processed images. 3D Slicer (www.slicer.org/), ITK-SNAP (www.itksnap.org/pmwiki/pmwiki.php), and MIM (www.mimsoftware.com/) are often used for segmentation of ROI (53). Third, we extract quantitative imaging features. Pyradiomics has now become a popular open-source Python package for extracting radiomic features from medical imaging (54). The primary categories of extracted radiomic features are shape-based features, histogram features (first-order features), texture features, and transform-based features. The shaped-based features describe the geometric properties of the tumor according to Shape-based (three-dimension) and Shape-based (two-dimension). In addition, first-order statistics describe the distribution of voxel intensities within the image region defined by the mask through commonly used and basic metrics. Texture features unfold the intra-tumoral heterogeneity. After resampling and filtering, transform-based features describe the frequency, spatial location, gray change, intensity, etc. Fourth, feature selection is performed on the extracted features using the filter, embedded or wrapper methods. Filter methods use statistics to rank and select the radiomic features, such as Pearson’s Correlation, t-test, Mann-Whitney U test, etc.; Wrapper methods use the chosen multi-variate model to evaluate and find the optimal radiomic features, such as Recursive Feature Elimination, Las Vegas Wrapper, etc.; Embedded methods embed radiomic features during modeling, and optimal features are selected by observing each iteration of the model training phase, such as Least Absolute Shrinkage and Selection Operator (LASSO), Ridge Regression, etc. Radiomic features correlating with tumor stage or gene expression can also be selected to evaluate their value for better prediction. The ultimate goal is to construct the targeted radiomic models, such as regression models, support vector machine (SVM), etc., to provide accurate stratification and assess their prognostic ability. After modeling, validation is usually evaluated through discrimination and calibration (55). The former, discrimination, refers to the performance that the radiomic model differentiates patients having a specific event at a different level of risk, and the latter, calibration, refers to the accuracy of absolute risk estimates. For accuracy of the performance in the radiomic model, bootstrap, cross-validation or hold-out methods are often utilized during discrimination and calibration. Bootstrap (or bootstrapping) is a uniform sampling method from a given training set. As a resampling technique, cross-validation employs various data subsets to test and trains a model over different iterations. The hold-out method divides the data into multiple segments, using one part to train the model and the rest to validate and test it. Noticeably, an internal or external validation set in the hold-out method may increase the reliability of the validation results for estimating its real diagnostic performance (**Figure 1**).

## 3 Neoadjuvant treatment

NAT is now one of the most commonly used treatments for cancer and has a wide range of clinical applications in the areas of pancreatic cancer, breast cancer, gastric cancer, colorectal cancer, and cholangiocarcinoma (56–59). To improve clinical prognosis and outcomes, NAT has also been introduced to the treatment of EC, especially for patients with locally advanced EC. The primary neoadjuvant therapies (NATs) for EC are neoadjuvant chemotherapy (nCT), neoadjuvant chemoradiotherapy (nCRT), and NAT combined with immunotherapy (60–62).

The British Medical Research Council (OE02) trial was the first large-scale study to demonstrate the survival benefits of nCT for patients with EC (63). Also, several other studies made nCT one of the earliest standard treatments for locally advanced patients EC (64, 65). However, some studies indicated that perioperative chemotherapy regimens showed a survival benefit in distal esophageal and gastroesophageal junction adenocarcinoma, but only selected patients benefited from nCT vs. surgery alone for ESCC (66). The clinical application of nCT is still investigated in further trials.

According to several landmark trials, nCRT is superior to surgery alone in some aspects, including R0 resection, survival outcomes and recurrence, which provides excellent clinical utility (5, 67, 68). The AGITG DOCTOR trial also showed that offering second-line chemotherapy and radiation improved survival for patients who did not respond to initial chemotherapy (69). And the chemoradiotherapy for EC followed by surgery study (CROSS) trial demonstrated a survival benefit compared to surgery alone when using chemoradiation with the addition of paclitaxel (68).

NAT combined with immunotherapy has developed rapidly in recent years, achieving sound therapeutic effects in various cancer treatments. Previous studies have shown its potential therapeutic effect (70, 71). A meta-analysis enrolled 759 patients from 21 studies using the major pathologic response and pCR to evaluate the effectiveness of nCT combined with immunotherapy (72). Of the enrolled patients, major pathological remission was achieved in 52.0% (95% CI: 0.44-0.57) of patients on nCT combined with immunotherapy, and pCR was achieved in 29.5% (95% CI: 0.25-0.32) of patients.

Despite the widespread use of NAT in clinical practice, some drawbacks are hard to predict, including harmful toxic effects, outdated technology, and failure to address patients’ and hospitals’ actual requirements (25, 73). Its future development still depends on individual characteristics and hospital technology, such as physical condition, pCR or recurrence prediction, and more multidisciplinary combination therapy (61). Noticeably, based on accurate assessment and prediction, the application of radiomics may help to reduce these deficiencies and prevent further complications of NAT in EC.

## 4 The application of radiomics for predicting the efficacy after NAT

### 4.1 Pathological complete response

pCR is defined as the absence of disease in the resected specimen’s esophagus and lymph nodes (T0N0). For patients with locally advanced EC, it has been correlated with a better outcome than non-pCR, which means there may be better survival and a lower local recurrence rate, providing a much better quality of life (74, 75). In this context, many techniques based on radiomics can be utilized to construct prediction models for pCR in EC patients after NAT, offering a bright prospect.

First, the CT-based radiomic model to predict pCR after NAT has a good prediction effect, especially in ESCC patients, with a high-performing level and good discrimination ability. Yang et al. (24) reported that three CT-based radiomic models could predict pCR in ESCC patients after nCRT in both the training (AUC, 0.84-0.86) and test cohorts (AUC, 0.71-0.79). In addition, peritumoral features can also serve as powerful prognostic indicators to construct radiomic models. Based on intratumoral and peritumoral features, Hu et al. (26) found that the combination of the two to establish a joint CT-based radiomic model had good identification performance and better prediction of pCR. There are also a small number of studies with general prediction results, which may be due to unestablished measurement errors, inconsistent standards, poor actual imaging quality, and small sample size (27). These aspects need to be explored further and improved in future research.

It is noteworthy that an increasing number of studies have also linked the radiomic features of PET alone or PET/CT to pCR. Previous studies have found that combining clinical factors and 18F-FDG PET-based radiomic features improves the ability to predict pCR (28). Meanwhile, CT can make up for the low anatomical spatial resolution of PET and provide more abundant radiomic features. Therefore, more PET/CT-based radiomic models are used to predict pCR after NAT in EC patients. PET/CT-based radiomic studies improved the predictive ability of pCR compared with PET alone and CT alone (AUCs for CT, PET, and PET/CT models were 0.73 ± 0.08, 0.66 ± 0.08, and 0.77 ± 0.07, respectively) (29). Beukinga et al. (30) constructed five different response prediction models based on eighteen clinical, geometric, and pre-processed texture features that were finally selected in PET and CT imaging. The predictive values were better than those of the models based on maximum standardized uptake values, demonstrating the advantages of PET/CT radiomic features over traditional parameters. SVM and logistic regression (LR) models can also be further constructed to predict the pathological response of tumors to nCRT. Lin et al. (75) reported that the SVM model obtained high accuracy (AUC, 1.00) and precision (no error classification), which was significantly better than traditional PET/CT measurements or clinical parameters. In general, using complementarity between imaging techniques such as PET/CT can effectively supplement radiomic features, further establishing a more accurate prediction model.

Moreover, diffusion-weighted magnetic resonance imaging (DW-MRI) has proven its value in predicting pCR in EC after NAT. A study by Borggreve et al. (31) was conducted to determine the optimal timing of DW-MRI for predicting pCR to nCRT for EC. The relative change in tumor apparent diffusion coefficient (ΔADC(%)) during the first two weeks of nCRT is the most predictive for pCR to nCRT in EC patients. They found that a model including ΔADC_week 2_ could discriminate between pathologic complete responders and non-pathologic complete responders in 87%. 18F-FDG PET/CT and dynamic contrast-enhanced magnetic resonance imaging (DCE-MRI) have also been used to predict pCR after nCRT in patients with locally advanced ESCC. Integrating 18F-FDG PET/CT and DW-MRI parameters can more accurately identify the pathological response of ESCC primary tumors to nCRT, especially the related prediction of pCR (AUC, 0.914) (76).

In addition to clinical and metabolic parameters, radiomic features combined with biological expression products can also improve the accuracy of radiomic models. Biological expression products such as the cluster of differentiation 44 (CD44) and the hedgehog (HH) signaling pathway ligand Sonic Hedgehog (SHH), which are closely related to the prognosis of EC patients treated with nCRT, can be included in the comprehensive prediction model (77). Beukinga et al. (78) included human epidermal growth factor receptor 2 (HER2) and CD44 in the clinic-radiomic model, which improved the overall performance of the nCRT response in EC patients (AUC, 0.857), thus facilitating the differentiation of pCR.

Therefore, it is urgent to accurately predict the pCR of EC patients, especially for patients with different NATs (66). Some studies have also found that predicting pCR based on the pathological subtypes of patients can improve the performance of radiomic models, especially in ESCC patients relative to EAC patients. The potential mechanisms may be the difference in pCR rate and genomic characteristics (33, 79). In summary, radiomic studies for predicting pCR in patients with EC after NAT have broad prospects, and their clinical application is worthy of further exploration.

### 4.2 Recurrence

A previous study reported that preoperative use of NAT, such as nCRT, can reduce recurrence rates in EC patients (68). Although researchers have provided recent advances in prognostic stratification and modern multimodal treatment strategies, many EC patients still have a tumor recurrence and eventually die of the disease, mainly in the distance (32, 80–82). Therefore, developing a more accurate prediction model for recurrence in EC patients is necessary. As an emerging non-invasive method, the radiomics-based prediction model can be a helpful tool to accurately predict the recurrence in EC patients after NAT and has a similar effect to pCR.

The radiomic methods used to predict recurrence are mainly carried out through PET/CT. 18F-FDG PET/CT has been demonstrated to be an accurate and indispensable imaging technique in the diagnosis and staging of EC, and it is the most useful method for detecting asymptomatic recurrence in patients undergoing curative treatment for EC (36). During the follow-up of a study by Chang et al. (37), higher values of 18F-FDG PET/CT parameters were associated with poor recurrence-free survival (RFS). Radiomics-based prediction methods can predict RFS and other indicators and, thus, reflect the recurrence situation. In another study to predict the prognosis of EC patients after nCRT, all patients underwent 18F-FDG PET/CT before and after nCRT (32). Pretreatment radiomic features and changes in the PET-derived traditional parameters after nCRT were analyzed, and recurrence was also well predicted. Additionally, the composite radiomic features from pretreatment non-contrast CT and staging PET are highly accurate in predicting response in EC, especially recurrence (34). In short, the current studies have shown the value of methods based on radiomics in predicting recurrence in EC patients after NAT. In particular, the predicting model based on PET/CT radiomic research has excellent advantages.

In addition, few studies have investigated the prediction of recurrence in patients achieving pCR. In EC patients, pCR after nCRT is accompanied by a lower rate of recurrence and more prolonged survival than non-pCR (29). Hence, predicting the likelihood of recurrence in these patients is still important, ensuring that an appropriately tailored treatment strategy is implemented early in the cohort of patients with a high risk of recurrence (35). Studies based on radiomics to predict the risk of recurrence after NAT in EC patients who achieve pCR are underway. A radiomic nomogram incorporating radiomic features and clinical factors has been developed and can be used in postoperative assessments of the individual recurrence risk in patients achieving pCR (35). Comparing the radiomic signature (*P* < 0.001) and clinical nomogram (*P* < 0.001) in both the training (AUC, 0.746 vs. 0.685 vs. 0.614, respectively) and validation cohorts (AUC: 0.724 vs. 0.671 vs. 0.629, respectively), an improved ability to predict the postoperative recurrence risk in patients with ESCC who achieved pCR after nCRT followed by surgery has been shown. However, further research based on radiomics is required to predict recurrence in patients who eventually achieve pCR.

Therefore, the value of using radiomics to predict the recurrence of EC patients after NAT has been proven whether recurrence occurs after pCR. This promising and developing prediction method still needs to be further studied in the future to predict post-NAT recurrence in EC patients more accurately.

### 4.3 Survival

Survival of EC patients can generally be improved with NAT, but there is still the possibility of some risk factors that could seriously affect the survival prognosis. Thus, a predictive survival model in EC patients after NAT is necessary. In recent years, radiomic analysis has been proven effective in predicting tumor treatment response and patient survival (29, 38). Better survival can be implied if radiomics can anticipate the emergence of pCR following nCRT (75). Moreover, a radiomic model that primarily relies on PET, CT, and MRI data can be utilized to forecast the survival of EC patients after NAT.

As a suitable method, PET can help predict the survival of EC patients after NAT. The combination of traditional PET parameters and radiomic parameters is effective in predicting the survival of ESCC patients. Patients can be more effectively grouped into subgroups with different survival rates by combining the conventional and radiomic parameters of 18F-FDG PET with clinical analysis, which is beneficial for further treatment (32).

Another valuable tool for estimating EC patients’ survival is the CT-based radiomics model. A study based on CT by Ruben et al. (83) developed and externally validated a random forest (RF) model using pretreatment CT radiomic features to predict 3-year overall survival (OS) in EC. The radiomic model had better predictive capability than the model using standard clinical variables (AUC, 0.69 vs. 0.63). The study by Lu et al. (84) found that, compared with the clinical nomogram, the radiomic-clinical nomogram improved the calibration and classification accuracy for OS prediction with a total net reclassification improvement of 26.9% (*P* = 0.008) and integrated discrimination improvement of 6.8% (*P* < 0.001). The results also concluded that based on CT, integrating the dual-region radiomic signature and clinicopathological factors improves OS prediction.

Additionally, researchers found that a combination of PET and CT was beneficial for predicting the survival of EC patients after NAT. The metabolic tumor volume (MTV) parameters measured by 18F-FDG PET/CT can also predict OS and RFS in patients with locally advanced EC (37). In addition, using an RF classifier based on 18F-FDG PET can also improve predictive and prognostic values, such as OS and RFS, compared to traditional survival analysis when applied to several tens of features in a limited database (85).

Furthermore, MRI is an excellent resource for creating predictive models. DCE-MRI and DW-MRI have been shown to have encouraging effects in predicting tumor response to nCRT and patient survival (86, 87). An MRI-based radiomic study also found that ADC skewness (AUC, 0.86) was the most useful ADC-derived parameter for predicting pCR and survival in ESCC patients receiving preoperative CRT therapy, which also confirms the feasibility of MRI-based radiomics in predicting survival (43). Notably, combining the individual and combined values of 18F-FDG PET/CT and DW-MRI during and after nCRT can validate the value of different radiomic approaches combined to predict survival (39).

Hence, some methods based on radiomics can predict the survival of EC patients after NAT, especially PET, CT and MRI. Future studies should focus on the continued optimization of predictive models, such as the relationship between pCR and survival. More informative radiomic features related to accurate survival prediction should be explored while better techniques such as artificial intelligence and deep learning can be utilized, which can be applied to optimize the screening of radiomic features (27, 88).

## 5 Discussion and suggestions

Radiomics has shown promising results when used to predict post-NAT responses in EC, particularly in predicting pCR, recurrence, and survival. However, the practical applications of radiomics still have some restrictions because of numerous factors (44). The primary sources of variability and pitfalls in radiomic research are study design, image acquisition and processing, and statistical analysis (89). In addition, some general defects in radiomic studies also impact their reliability and practical application. Thus, this article summarizes the following viewpoints to provide valuable solutions and possible directions for future research on radiomics in predicting the efficacy of patients with EC after NAT.

Radiomic analysis will be affected by the systematic errors of research design, resulting in its defects and deficiencies. Incorporation bias and spectrum bias can often be found (89). The outcome of using data from the analyzed images caused the incorporation bias. Defining the outcome from the analyzed image should be avoided. And spectrum bias is from models developed using only extreme cases, which means that researchers must ensure study data are generalizable to the population of interest.

Importantly, standards of radiomics must be established and further refined among different suppliers and institutions, promoting the standardization of radiomic research and improving its practical application (88, 90). Moreover, image acquisition and processing reasons include software and operator variability (89). Software variability means that hand-engineered features, calculated using a different software platform or version of the same software, may have different values despite adhering to accepted standards. The operator variability is caused by manual or semi-automatic delineation of ROI, so ROI should be scrutinized by experienced physicians or reduce and correct variability in ROI.

Additionally, there are still some improvements in the process before and during statistical analysis. First, imaging professionals should continue improving imaging quality and the method of delineating the ROI, because tumor segmentation could be challenging for small lesions (91), and the extracted radiomic features may raise the question of repeatability (29, 76). For instance, applying pre-processing before image analysis can optimize the performance of models, and proper feature selection methods can reduce the dimensionality of the generated data (92). Bias from overfitting, optimistic performance bias, and bias from the exclusion of indeterminate or missing feature data are often found in many radiomic research (89, 92). Researchers can evaluate the model on an independent external data set and use resampling methods, such as cross-validation, to decrease these biases as possible.

In many radiomic studies, some mutual deficiencies leading to unreliability and non-repeatability of their results should be solved. First, an increasing number of prospective, multicenter, large simple studies with external validation are needed. Currently, most of the studies were performed retrospectively, which means bias generated from the retrospective review could not be avoided (32, 37). Although limited resources restrict the development of multicenter prospective studies, their importance cannot be overemphasized. Borggreve et al. (39) conducted a multicenter prospective study to evaluate the individual and combined value of 18F-FDG PET/CT and DW-MRI. They found that changes in 18F-FDG PET/CT after nCRT and early changes in DW-MRI during nCRT contributed to the identification of nCRT by pCR in EC. Researchers also found that 18F-FDG PET/CT and DW-MRI may have complementary value in the evaluation of pCR, which is consistent with previous research results (76). Simultaneously, large sample sizes and rich external validation are also required to verify the accuracy of prediction models (27, 30, 39, 76). Second, the study of targeted radiomic prediction techniques is urgently needed for various NATs (66). Third, the links between radiomics and other disciplines deserve further strengthening; one example that has achieved good results in recent years is radio-genomics, in which it is assumed that imaging features are related to gene signatures (44). Multimodal technology has also proven its benefits, which combine multiple imaging techniques. PET/CT combined with MRI, is proven its benefits for predicting models (39, 76).

At present, the application of radiomics to predict the efficacy after NAT has become a popular and essential direction for patients with EC. In the future, applying radiomics in EC will be conducive to improving post-NAT efficacy prediction providing timely and accurate treatment strategies that truly benefit EC patients.

## Author contributions

HG led the other two co-first authors, H-TT and W-LH, in manuscript preparation, reference literature review, and manuscript writing. J-JW, P-ZL, J-JY, HH, and H-JY were responsible for collection and sorting of literature. S-LH, Y-JZ, Z-QD, K-YJ, and X-YZ revised the manuscript. DT and H-NZ are responsible for the critical revision of the manuscript. All authors contributed to the article and approved the submitted version.
